# Encapsulation of Aconitine in Self-Assembled Licorice Protein Nanoparticles Reduces the Toxicity In Vivo

**DOI:** 10.1186/s11671-015-1155-1

**Published:** 2015-11-19

**Authors:** Li-jing Ke, Guan-zhen Gao, Yong Shen, Jian-wu Zhou, Ping-fan Rao

**Affiliations:** Food Nutrition Sciences Centre, Zhejiang Gongshang University, Room 407, No. 1 Laboratory Bld., No. 149 Jiaogong Road, Xihu District, Hangzhou, 310012 Zhejiang Province China; Institute of Biotechnology, Fuzhou University, No. 523 Gongye Road, Gulou District, Fuzhou, 350002 China

**Keywords:** *Radix glycyrrhizae*, *Radix aconite lateralis preparata*, Protein nanoparticles, Aconitine encapsulation, Toxicity reduction

## Abstract

Many herbal medicines and compositions are clinically effective but challenged by its safety risks, i.e., aconitine (AC) from aconite species. The combined use of *Radix glycyrrhizae* (licorice) with *Radix aconite L.* effectively eliminates toxicity of the later while increasing efficacy. In this study, a boiling-stable 31-kDa protein (namely GP) was purified from licorice and self-assembled into nanoparticles (206.2 ± 2.0 nm) at pH 5.0, 25 °C. The aconitine-encapsulated GP nanoparticles (238.2 ± 1.2 nm) were prepared following the same procedure and tested for its toxicity by intraperitoneal injection on ICR mouse (*n* = 8). Injection of GP-AC nanoparticles and the mixed licorice-aconite decoction, respectively, caused mild recoverable toxic effects and no death, while the aconitine, particle-free GP-AC mixture and aconite decoction induced sever toxic effects and 100 % death. Encapsulation of poisonous alkaloids into self-assembled herbal protein nanoparticles contributes to toxicity attenuation of combined use of herbs, implying a prototype nanostructure and a universal principle for the safer clinical applications of herbal medicines.

## Background

Aconitine (AC), known as devil’s helmet, is a highly poisonous alkaloid derived from various aconite species. Aconitine and its derivatives are the major effective but also toxic compositions of a Chinese medicinal herb, lateral roots of *Aconitum carmichaelii* Debx (*Radix aconite lateralis*, Sichuan aconite root) [1, 2], and an Indian herbal medicine, roots of *Aconitum heterophyllum* Wall (Atis) [3]. Including more than 300 species worldwide, the plants from genus *Aconitum* are used for treating diseases or ailments such as rheumatic arthritis, cold, and pain [3]. Despite its cardiotoxic and neurotoxic risks, aconitine is popularly used as the antipyretic and analgesic agents in many Asian countries. It is difficult to calculate appropriate dosage for the use of aconitine due to its narrow therapeutic index [4].

As an essential measure for clinical practice of traditional Chinese medicine, it is recommended that the aconite roots shall be used together with roots of *Glycyrrhiza uralensis* Fisch (*Radix glycyrrhizae*, Gan-Cao, licorice root) to eliminate the toxicity and improve efficacy [5, 6]. It has been known that liquiritin from licorice forms complexation with aconitine and therefore reduces the amount of free aconitine [7], indicating that interactions between aconitine with other major amphiphilic compounds (i.e., protein) from licorice may facilitate the formation of aconitine complex of such kind, too.

Micro/nano-structures composed of self-assembled protein have been studied with proteins from different natural products, i.e., whey protein, casein, soy protein, and zein, for their potential as efficient and safe carriers for nutrients and drugs [8, 9]. When exposed to biological medium or multiple composition dispersions like herbal decoction, protein corona formed on the nanoparticles (NPs) diversely alters the biological fate and pharmaceutical efficacy of particles [10]. The intracellular protein extracts of a fungal strain *Pycnoporus sanguineus* was used as reducing and stabilizing agents to synthesize AuNPs with various shapes and dimensions, which present good catalyzing ability on the degradation of 4-nitroaniline [11].

Like many Chinese medicinal herbs, licorice root has high content of soluble proteins, some of which are glycated and remain soluble even in the boiling decoction. The ephedrine alkaloid-containing colloidal nanoparticles discovered in another licorice containing Chinese medicinal decoction [12] imply that licorice root proteins would hypothetically interact with aconitine to form aggregates thereafter affecting the toxicity. To elucidate this assumption, one of the major proteins from *Radix glycyrrhiza*, namely GP, was purified and used to construct NPs with aconitine embedded. Toxicity of the NPs was tested in vivo in comparison to pure aconitine, aconite, and licorice root decoction and reported here.

## Methods

### Materials

*Aconitum carmichaelii* Debx lateral root (*Radix aconite Lateralis*, Sichuan aconite root) and *Glycyrrhiza uralensis* Fisch., root and rhizome (*Radix glycyrrhizae*, Gan-Cao, licorice root) were purchased from Beijing Shuang-qiao-yan-jing Medicinal Material Factory, Co., Ltd. (Beijing, China) and authenticated by Prof. Chengzi Yang from Fujian University of Traditional Chinese Medicine. Voucher specimens were deposited at the Museum of Traditional Chinese Medicine, Fujian University of Traditional Chinese Medicine (Fuzhou, China), under the identification code: *Radix aconite Lateralis* (SQYJ-201303113SC6004) and *Radix glycyrrhizae* (SQYJ-201306062 NM1202). Aconitine (batch no. 20130525, HPLC purity >98.5 %) was provided by Fujian FDA, China.

### Preparation of Herbal Decoctions

Aconite lateral roots (30 g), licorice roots (30 g), and the equal mixture of both herbs (*w*/*w* = 30/30 g) were soaked in deionized water for 30 min and boiled for 30 min. The decoction was centrifuged at 12,000*g*, 25 °C for 15 min. The supernatants were collected and diluted to constant volume of 150 mL.

### Purification of Licorice Root Protein (GP)

The sundried licorice roots (200 g) were grinded into a fine powder and extracted with 20 mM phosphates buffer (pH 7.2, 100 mM NaCl) at a ratio of 1:5 (*w*/*v*) for 12 h at 4 °C. The extract was adjusted to pH 9.8–10 to remove glycyrrhizic acid. The crude protein extract was obtained by ethanol precipitation (40~60 %) and applied to liquid chromatographic isolations described as below: (1) Macro-Prep® High-Q anionic exchange chromatographic column (10 × 250 mm): pre-equilibrated with 20 mM Tris–HCl (pH 8.0); linear elution gradient 0~0.5 M NaCl, 200 mL; (2) POROS®R1 hydrophobic chromatographic column (10 × 250 mm), pre-equilibrated with deionized water, and eluted with linear 0~100 % acetonitrile gradient at 1.0 mL/min.

### Preparation of GP Nanoparticles (GP NPs) and GP-Aconitine Nanoparticles (GP-AC NPs)

The GP solution (0.46 mg/mL) was adjusted with 1 M HCl to pH 5.0 and placed at 20–25 °C for 10 min, for self-assembly of GP NPs. The GP-AC NPs were prepared by the same procedure with the addition of 10 μL methanol aconitine solution (20 mg/mL) in 1990 μL GP solution. The size and ζ-potential of particles were characterized with dynamic light scattering analysis (DLS) on a Zetasizer Nano device (Malvern Instruments, Worcestershire, UK) and size-exclusive HPLC (TSK gel G6000PW, 7.8 mm × 30 cm, 20 mM pH 7.2 phosphates buffer at 0.5 mL/min, 280 nm UV detection).

### Protein SDS-PAGE

The GP was analyzed with standard SDS-PAGE in 4 % concentrating gel and 12.5 % separating gel and stained with Coomassie brilliant blue G-250 according to Laemmli method [13], with a protein gel electrophoresis device (ATTO, Japan).

### Protein Content Determination

The protein concentration was determined with Folin-phenol assay [14] with bovine serum albumin as standard sample.

### Scanning Electron Microscopy (SEM)

SEM samples were prepared according to Rudiger’s method [15], the nanoparticles suspension was gently collected with 0.22 μm cellulose acetate membrane, fixed and dehydrated, and dried and coated with gold. The NPs on the membrane was then imaged with a Cold Field Emission S-4800 Scanning Electron Microscope (Hitachi, Tokyo, Japan) operated under an acceleration voltage of 5 kV. The images were taken with 100,000 (500-nm scale bar) and 25,000 magnification (2-μm scale bar).

### Quantification of Aconitine

The content of AC was determined with reversed-phase liquid chromatography (RPLC) column Eurospher 100-5 C18 (250 × 4.6 mm, 5 μm), eluted with a gradient of solution A (acetonitrile–tetrahydrofuran, 25:15, *v*/*v*) and solution B (0.1 mol/L ammonium acetate, 0.5 mL/L glacial acetic acid) [16, 17]. The percentage of solution A raised from 15 to 20 % in 40 min and stayed at 20 % for 10 min. Flow rate was 1.0 mL/min, UV detector wavelength at 250 nm, and column temperature at 35 °C. The AC concentration was calculated according to the standard curve.

### Encapsulation Rate of Aconitine in GP-AC NPs

The suspension of GP-AC NPs was filtrated with ultrafiltration tubes (MW cutoff 100 kDa). The AC content of the filtrates was determined with the above method. The encapsulation rate was calculated with equation below.

$E=\frac{C-{C}_1}{C}$ (C: total AC in suspension; C1: AC in filtrates)

### Mouse Acute Toxicity Test

The herbal extracts (0.4 mL/mice) and GP-AC NPs suspension/solution (0.2 mL/mice) were given to healthy ICR mouse (weight 15 ± 1.0 g, eight per group) by intraperitoneal injection (as shown in Table 1). The toxic response was recorded 3 h after the injection [18].

**Table 1:** Groups in acute toxicity tests in mice by intraperitoneal injection

Group	Mouse	Samples and dosage (per mice)
A	8	Licorice decoction (30 g licorice), 0.4 mL (AC = 0 μg)
B	8	Aconite decoction (30 g aconite), 0.4 mL (AC = 8.0 μg)
C	8	Licorice + aconite decoction (30 g licorice, 30 g aconite), 0.4 mL (AC = 1.2 μg)
1	8	Tris–HCl buffer pH 5.0, 0.2 mL
2	8	Aconitine dissolved in Tris–HCl buffer (pH 5.0), 0.2 mL (AC = 10.0 μg)
3	8	GP-AC colloidal dispersion contains NPs, GP, AC, 0.2 mL (AC = 10.0 μg)
4	8	Filtrates solution contains GP and AC, 0.2 mL (AC = 10.0 μg)
5	8	GP-AC NPs dispersion, 0.2 mL (AC = 10.0 μg)

## Results and Discussion

### Results

#### Purification of the Major Protein from Radix Glycyrrhizae

**Fig. 1:**
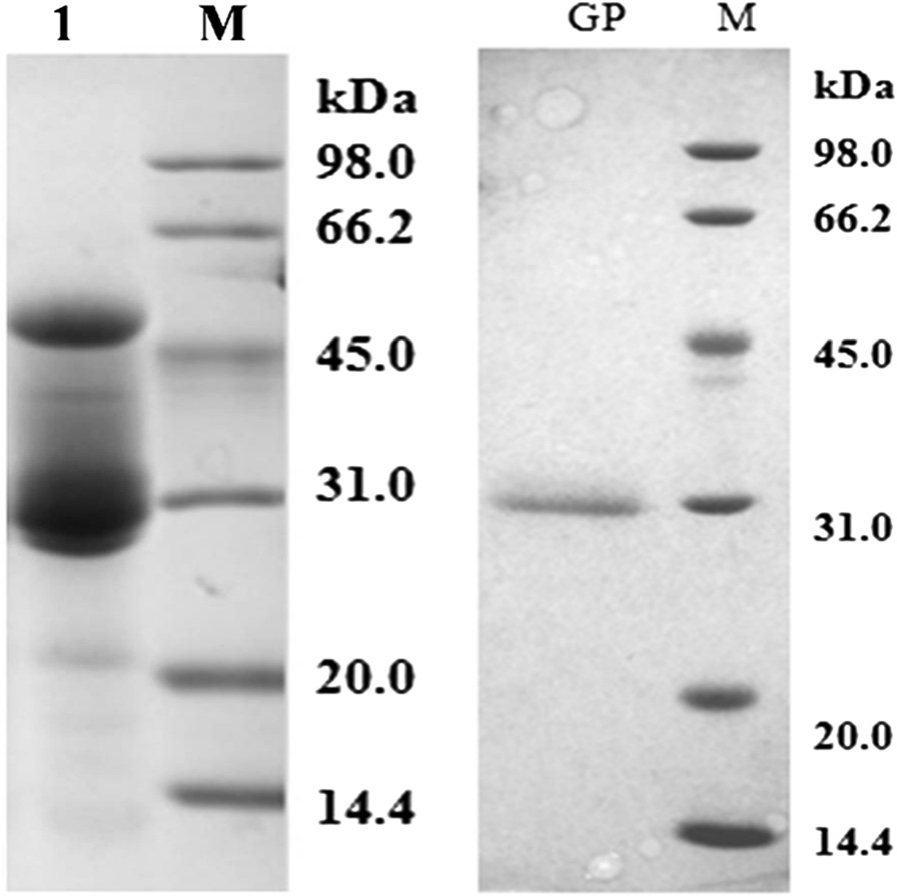
SDS-PAGE of *Radix glycyrrhizae* proteins. Samples: *M* marker; *Lane1*, crude extract of *Radix glycyrrhizae*; *GP*, licorice protein. Electrophoresis was performed at following conditions: 12.5 % gel, 120 V, 25 μL/sample, stained with Coomassie brilliant blue. Equation for molecular weight calculation: sample protein MW = −0.9231 × migration distance + 5.0148 (*R* ^2^ = 0.9994)

The licorice proteins were extracted from the sun-dried *Radix glycyrrhizae* and firstly separated by ethanol precipitated. As shown in Fig. 1, the crude extracts contain proteins with molecular weights ranged from 14.0 to 66.2 kDa. Among which the protein with molecular weight around 31.0 kDa has the highest content and remained in the decoction of licorice. This protein was purified with anionic exchange liquid chromatography (High-Q), followed by hydrophobic liquid chromatography (POROS® R1), and named as GP. The purity was certified with SDS-PAGE as shown in Fig. 1. The PI of GP was 4.5 determined with gel electrophoresis.

**Fig. 2:**
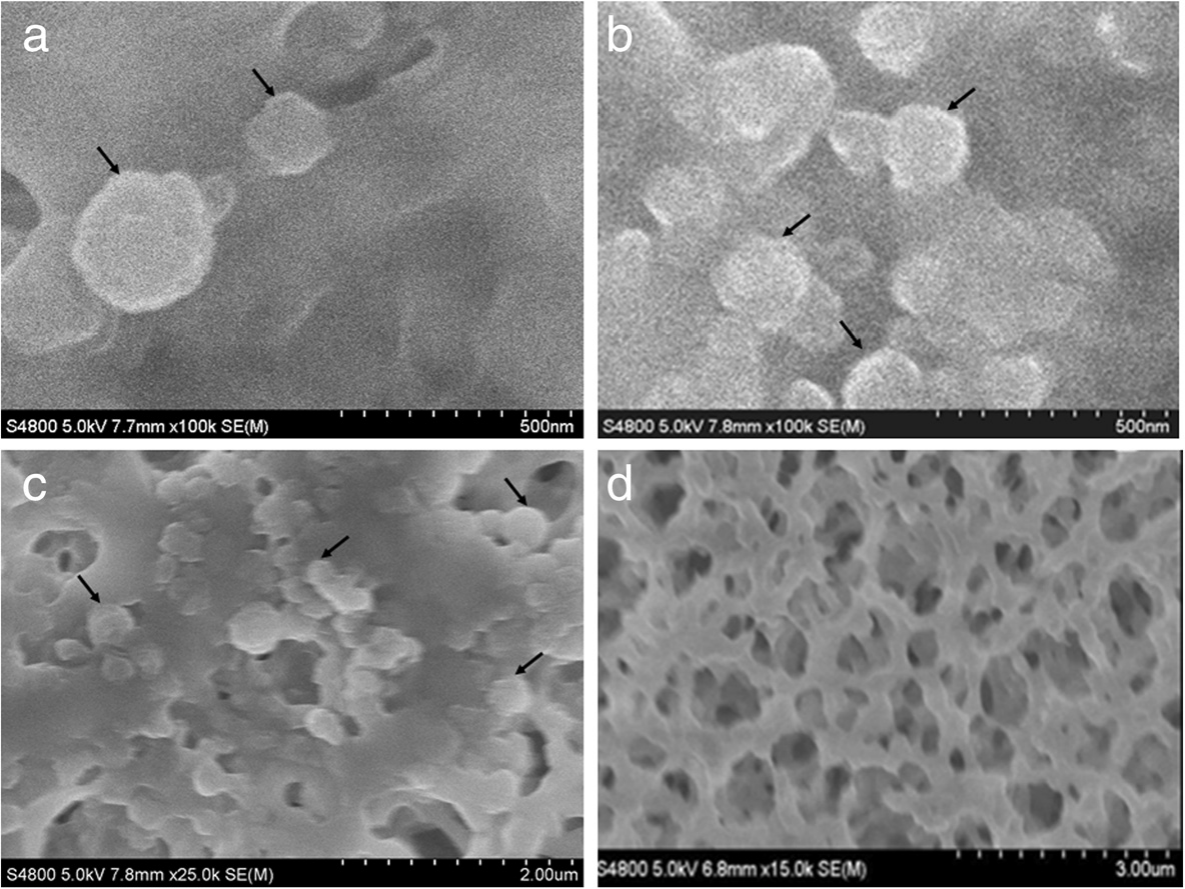
SEM image of licorice protein (*GP*) nanoparticles. GP NPs were presented on the top of cellulose acetate membrane. **a, b** GP NPs, amplified 100,000 times. **c** GP NPs, amplified 25,000 times. **d** empty cellulose acetate membrane

**Fig. 3:**
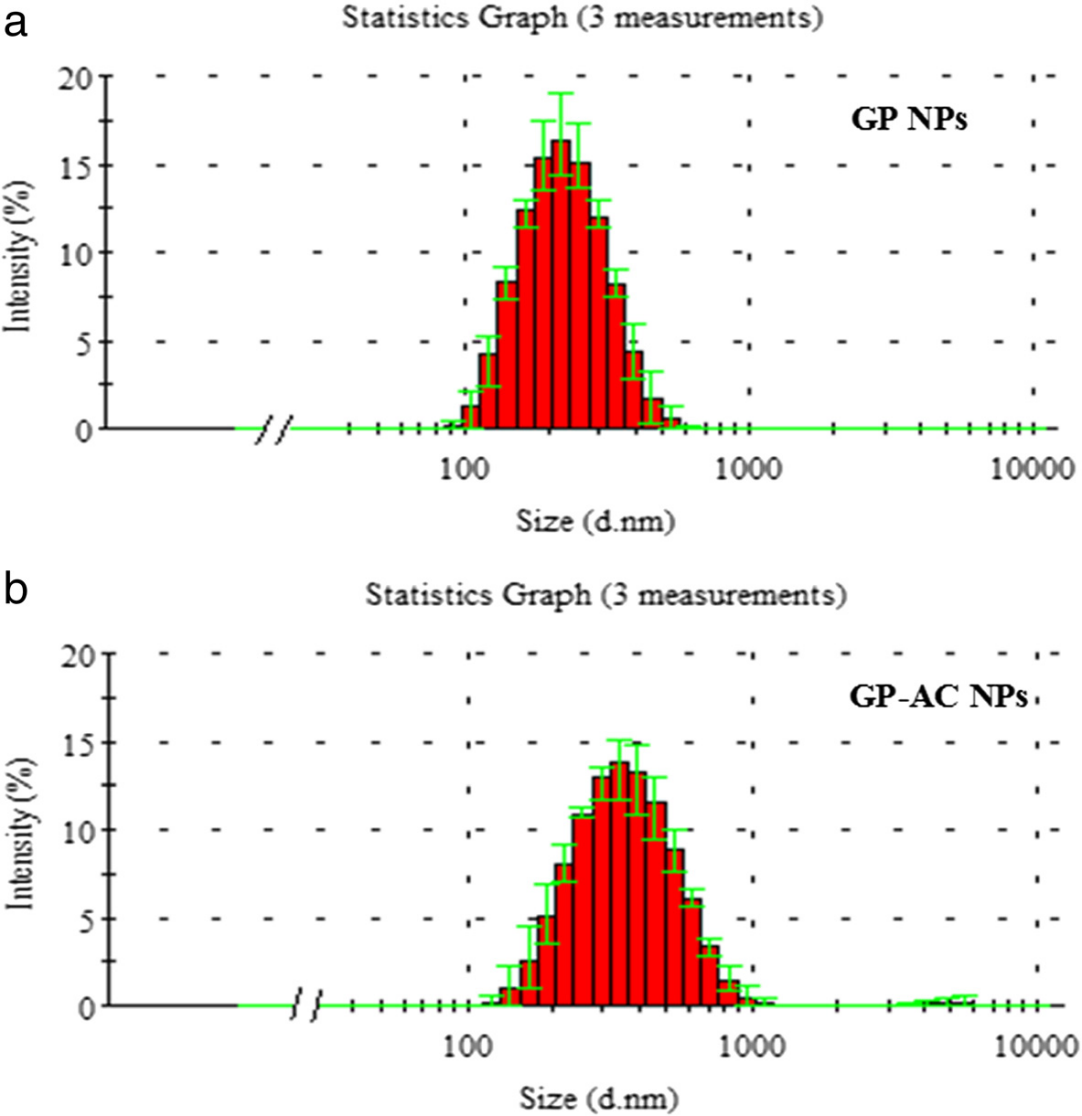
Size (diameter) distribution of GP NPs and GP-AC NPs. **a** GP NPs diameter distribution; **b** GP-AC NPs diameter distribution

Either GP alone or mixture of GP and AC self-assembled into near-spherical colloidal nanostructures (as shown in Fig. 2) at pH 5.0, at protein concentration of 460 μg/mL and AC content of 100 μg/mL. The average diameter of GP nanoparticles (GP NPs) is 206.2 ± 2.0 nm (Fig. 3a), while the diameter distributes are from 100 to 500 nm. Addition of AC increased the mean diameter of particles, which is 238.2 ± 1.2 nm, and extended the diameter distribution of particles up to 900 nm.

#### Quantification of AC Content and Calculation of Encapsulation Rate

The AC content of aconite decoction, mixed licorice-aconite decoction, GP-AC mixture, and ultrafiltration filtrates were quantified with RPLC method and regression equation of AC standard curve (*y* = 25,069*x* − 54,187, *R*^2^ = 0.9997).

There was 93.89 μg/mL AC in GP-AC mixture before ultrafiltration whereas 67.39 μg/mL AC in filtrates; the implied 28.2 % of AC was encapsulated into the GP-AC NPs. Given that the protein content in GP-AC NPs was 244.6 μg/mL, determined with Lowry assay, the molecular weight ratio of GP/AC in the NPs was 9.2/1, which is equal to 1:5.2 in molar ratio. For reference, the AC contents of aconite decoction (group B) and mixed licorice-aconite decoction (group C) used in the toxicity test were 20.1 and 3.1 μg/mL, respectively.

**Fig. 4:**
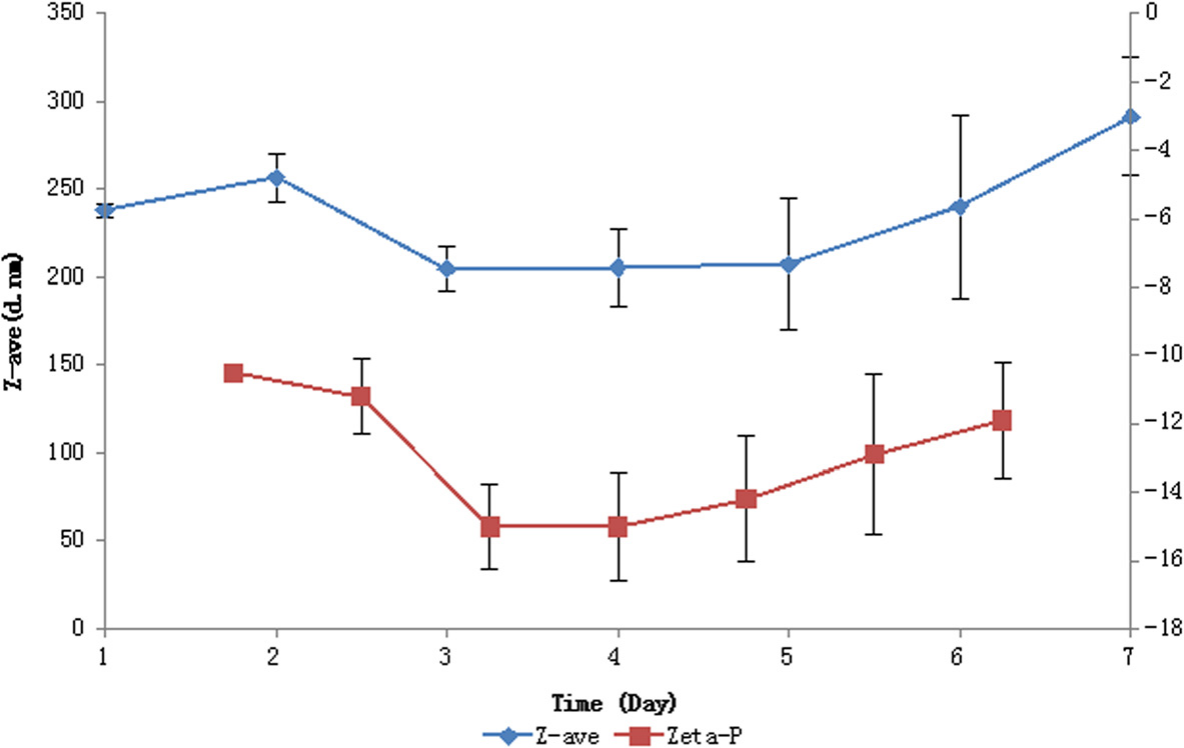
GP-AC particle size and ζ-potential variation over time at 25 °C. *Z*-average diameter of GP-AC NPs (*diamond*), ζ-potential of GP-AC NPs (*square*). Three duplicates were performed to calculate the average value and variations

The GP-AC NPs remained rather stable at 25 °C, as indicated by the constant monitoring of particle size (diameters) and ζ-potential for 7 days (Fig. 4). The average diameter increased mildly by 50 nm (<25 %) after 7 days, while the variation of which increased along the extension of time. The ζ-potential during the period fluctuated slightly but stayed in a narrow range between −10 and −16 mV, with an increased variation. There were no visible aggregates or precipitates formed during the period of observation, indicating a potential of GP NPs as prototype of drug vehicle.

#### Acute Toxicity Tests

Two lines of samples were tested for the acute toxic response of aconitine: (1) the herbal decoctions including licorice roots, aconite roots, mixed licorice, and aconite roots (*w*/*w* = 1:1); (2) aconitine solution, GP-AC mixture dispersion at pH 5.0, filtrates, and cutoff aggregates (GP-AC NPs) of the mixture separated by ultrafiltration (MW cutoff 100 kDa). AC contents of the second line samples were adjusted to 50 μg/mL to give a universal substance background for toxicity comparison.

As shown in Table 2, after intraperitoneal injection, no toxic effects were observed among mouse received licorice decoction (group A), while mouse received aconite decoction (group B) exhibited severe toxic responses and eventually all died at approximately 100 min after injection. In comparison, no death was reported among mouse received mixed licorice-aconite decoction, while mild toxic effects were observed but subsided in 3 h.

**Table 2:** Acute toxicity tests in mice by intraperitoneal injection (*n* = 8)

Group	Dead	Time to death (min)	Toxic effects
a	0	–	Normal
b	8	100 ± 15	Gather, eyes close, tremble, convulsions, opisthotonos position
c	0	–	Gather, eyes close, less active, returned to normal in 3 h
1	0	–	Normal
2	8	18 ± 5	Gather, eyes close, tremble, convulsions, opisthotonos position
3	8	30 ± 5	Gather, eyes close, tremble, convulsions, opisthotonos position
4	8	50 ± 5	Gather, eyes close, tremble, convulsions, opisthotonos position
5	0	–	Gather, eyes close, less active, returned to normal in 3 h

Mouse received aconitine solution (group 2) exhibited severe toxic effects of aconitine and died 18 min after injection. Mouse received GP-AC colloidal dispersions (containing NPs, GP, AC; group 3), and filtrates from ultrafiltration (containing free GP and AC; group 4) exhibited severe toxic effects, too, and died 30 and 50 min after injection, respectively. The death time of mouse received GP-AC mixtures were delayed compared to the pure aconitine group. The mouse administrated with particulates trapped by the membrane (GP-AC NPs and GP NPs; group 5) only exhibited mild toxic response to and all subsided in 3 h and survived.

## Discussion

The encapsulation of AC into GP NPs increased the mean particle diameter by 16 % in comparison to the particles of GP alone. Similar size expanding was observed in doxorubicin encapsulated BSA nanoparticles [19]. The binding force between protein and small molecule phytochemicals generally involves either ionic interaction, hydrogen bonds, or hydrophobic force [20]. The water soluble compound like ephedrine attached to the surface of protein nanoparticles via secondary boundary [12], while the water insoluble compounds like aconitine is more likely to be integrated inside the protein nanoparticles through boundary with hydrophobic domain of protein. The former kind has a loose boundary to the particles and is easy to be released when the suspension environment become less polar (more nonpolar), i.e., the increase of methanol concentration. The later kind forms a much more stable supramolecular structure and is hardly broken by changing the polarity of solvent, i.e., RPLC analysis. Ren et al. reported a good example of utilizing hydrophobic binding domains within protein (modified pyruvate dehydrogenase) nanostructure to retain and deliver the hydrophobic antitumor compound doxorubicin, with which a significant apoptosis was induced in breast cancer cells [21].

The decoction of mixed aconite and licorice (in group C) showed reduced toxicity in comparison to that of sole aconite, as shown in Table 2 (groups A/B/C). The aconitine content in aconite decoction was 20.1 μg/mL (group B), which dropped by 84 % to 3.1 μg/mL in the aconite-licorice decoction. The decrease of AC content provided an obvious reason to the decreased toxicity in group C, while other active compositions from licorice may have also contributed by suppressing the physiological impacts of aconitine, i.e., 6-benzoylheteratisine, as a natural antagonist of the Na^+^ channel activator aconitine [22]. These two factors may work individually or synergistically. Furthermore, the drop in AC content (group C) may be attributed to two possible reasons: the heating during herbal material processing and decoction preparation [3] or the complexation formation of aconitine with other compositions in the decoction, e.g., licorice protein in this study and liquiritin as reported [7]. We assume that the key reasons are more likely to be the complexation formation rather than heating, since the aconite decoction (group B) had gone through the same boiling process but retained higher concentration of AC.

**Fig. 5:**
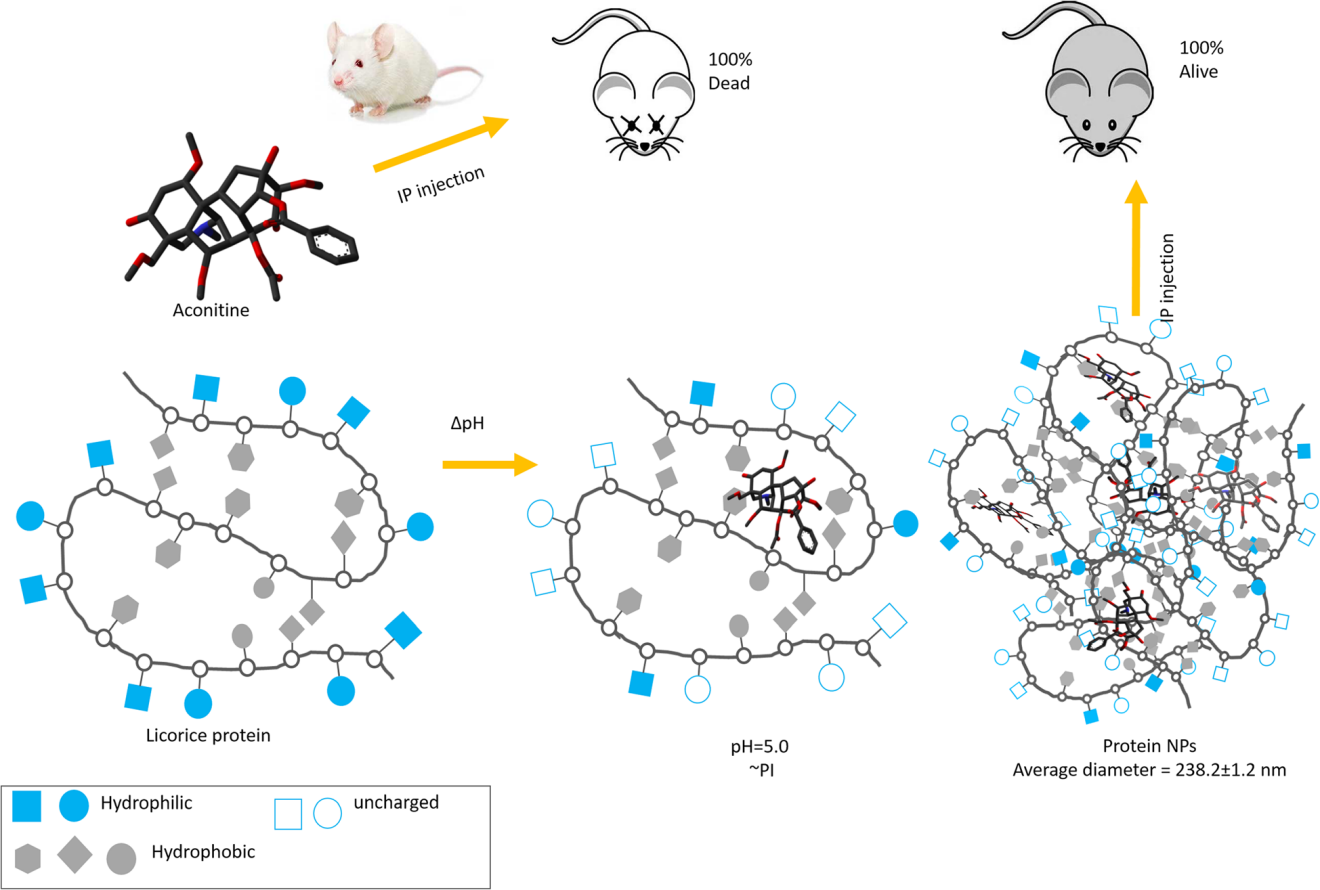
Schematic diagram of formation and detoxification of GP-AC NPs. The hydrophilic surface of GP molecules, taking a polypeptide segment as example (*bottom left* in the diagram), is charged (shown as *blue square* and *circular dot*) in the aqueous solution while the hydrophobic groups (shapes in *grey*) are wrapping inside the molecule. When the solution pH was adjusted to near PI (pH = 5.0), the protein surface became partially uncharged (shown as *hollow blue square* and *circle*) which weakens the ionic dispelling force between protein molecules and facilitates the protein aggregation. The water insoluble AC binds to the hydrophobic regions of GP during the conformational changes thereafter is encapsulated in the protein NPs, namely GP-AC NPs. The protein encapsulation detoxified AC in the mouse

The detoxification effects of GP encapsulation was accessed and demonstrated in healthy mouse at 50 μg/mL of aconitine, as shown in the schematic diagram (Fig. 5). In comparison to the 100 % death rate in pure aconitine group (group 2), GP-AC particles showed no vital consequence except some mild toxic response, i.e., gather, eyes close, less active (group 5), implying the very difference between the aconitine in single molecule form or in a supramolecular structure. The size, chemical composition, and surface properties of particulates determine the circulation and metabolism profiles of nanoscale drug carriers [19, 23]. Protein nanoparticle-based drug vehicle can improve the biocompatibility and absorption while reducing the toxicity, i.e., apotransferrin nanoparticles eliminated cardiotoxicity of doxorubicin when provided a longer bioavailability [24]. When the tumor cells and nonphagocytic cells tend to absorb relatively small particles (50 to 200 nm), NPs with such diameter and hydrophilic surface are able to escape from reticuloendothelial phagocytose [25, 26]. GP-AC NPs exceed 200 nm in diameters and is likely to be swallowed by phagocytes (macrophages) [26], kept out of blood circulation thereafter becoming less toxic.

Despite the similar toxic syndromes and eventual death, the survival minutes before death varied between groups 2, 3, and 4. The extended survival time (30 min) in group 3, which is a mixture of NPs, GP, and AC, may attribute to decline in the amount of free AC. As a mixture of free GP and AC in group 4, the survival time was unexpectedly extended further to 50 min. It may attribute to the secondary aggregation of GP and AC induced by the removal of GP-AC NPs from the otherwise balanced suspension.

## Conclusions

The encapsulation of aconitine in the pH-induced self-assembled licorice protein NPs eliminated the toxicity of aconitine in vivo. The study not only elucidates the self-assembled protein NPs contribute to the detoxifying effects of licorice against aconite but also provides a new approach and material for application of active phytochemicals and herbal compositions with much less safety concerns. On the other hand, the impacts of complexes among multiple compositions from the natural products should be considered in the toxicological evaluation, implying the appropriate approach and technique should be applied.

## Abbreviations

AC: aconitine
GP: the purified protein from licorice roots (*Radix glycyrrhiza*)
GP NPs: nanoparticles self-assembled with the protein
GP-AC NPs: nanoparticles assembled with the protein and aconitine
NPs: panoparticles
SEM: scanning electron microscope
